# Interactive effects of long-term management of crop residue and phosphorus fertilization on wheat productivity and soil health in the rice–wheat

**DOI:** 10.1038/s41598-024-51399-8

**Published:** 2024-01-16

**Authors:** Rajeev Kumar Gupta, Paramjit Kaur Sraw, Jasjit Singh Kang, Jagroop Kaur, Vivek Sharma, Neemisha Pathania, Anu Kalia, Nadhir Al-Ansari, Abed Alataway, Ahmed Z. Dewidar, Mohamed A. Mattar

**Affiliations:** 1 Department of Soil Science, Punjab Agricultural University, Ludhiana, Punjab 141004 India; 2 Department of Agronomy, Punjab Agricultural University, Ludhiana, 141004 India; 3 Department of Civil, Environmental, and Natural Resources Engineering, Lulea University of Technology, 97187 Lulea, Sweden; 4 Prince Sultan Bin Abdulaziz International Prize for Water Chair, Prince Sultan Institute for Environmental, Water and Desert Research, King Saud University, 11451 Riyadh, Saudi Arabia; 5 Department of Agricultural Engineering, College of Food and Agriculture Sciences, King Saud University, 11451 Riyadh, Saudi Arabia; 6 Agricultural Research Centre, Agricultural Engineering Research Institute (AEnRI), Giza, 12618 Egypt

**Keywords:** Plant sciences, Agroecology

## Abstract

In the context of degradation of soil health, environmental pollution, and yield stagnation in the rice–wheat system in the Indo-Gangetic Plains of South Asia, an experiment was established in split plot design to assess the long-term effect of crop residue management on productivity and phosphorus requirement of wheat in rice–wheat system. The experiment comprised of six crop residue management practices as the main treatment factor with three levels (0, 30 and 60 kg P_2_O_5_ ha^–1^) of phosphorus fertilizer as sub-treatments. Significant improvement in soil aggregation, bulk density, and infiltration rate was observed under residue management (retention/incorporation) treatments compared to residue removal or residue burning. Soil organic carbon (SOC), available nutrient content (N, P, and K), microbial count, and enzyme activities were also significantly higher in conservation tillage and residue-treated plots than without residue/burning treatments. The residue derived from both crops when was either retained/incorporated improved the soil organic carbon (0.80%) and resulted in a significant increase in SOC (73.9%) in the topsoil layer as compared to the conventional practice. The mean effect studies revealed that crop residue management practices and phosphorus levels significantly influenced wheat yield attributes and productivity. The higher grain yield of wheat was recorded in two treatments, i.e. the basal application of 60 kg P_2_O_5_ ha^–1^ without residue incorporation and the other with half the P-fertilizer (30 kg P_2_O_5_ ha^–1^) with rice residue only. The grain yield of wheat where the rice and wheat residue were either retained/incorporated without phosphorus application was at par with 30 and 60 kg P_2_O_5_ha^–1^. Phosphorus levels also significantly affected wheat productivity and available P content in the soil. Therefore, results suggested that crop residue retention following the conservation tillage approach improved the yield of wheat cultivated in the rice–wheat cropping system.

## Introduction

Rice–wheat cropping system is a predominant agricultural system followed in Asia, especially in South-eastern Asia covering an area of about 24 million hectares^1^. It occupies around 13.5 m ha spread over the Indo-Gangetic Plains of South Asia, of which India shares 10.5 m ha^2^. This cropping system is precarious for the livelihood, food security, employment, and income generation of millions of Asians, with a share of about 33 and 42% in total acreage under rice and wheat and a contribution of 25 and 33% in total rice and wheat production, respectively. The system is extensively practiced in southeastern countries, including the Indian peninsula. In India, it is the most prevalent cropping system in the northern states, with Punjab and Haryana contributing a principal share of 70% to the national food grain production. Straw generated from wheat cultivation is removed from the combine-harvested fields with the help of wheat straw combine for its utilization as animal feed. However, more than 80% of straw generated from rice fields is burnt because of the associated factors, viz., low economic value, labour scarcity, interference with the sowing of subsequent crops and short window period for managing rice straw before the sowing of wheat^3^.

The heat generated from burning further kills the environment-friendly useful soil microbes^4,5^ in addition to the release of greenhouse gases (GHGs) such as carbon dioxide (70%), carbon monoxide (7%), nitrous oxide (2.09%) and methane (0.66%)^6^. Unremitting the burning of these large amounts of crop residues causes a huge loss of nutrients and biodiversity, eventually raising the nutrient input cost and the depletion of long-term soil fertility and productivity^7^. Recent advancements in the field of crop residue management have developed numerous residue management options, viz. surface retention of rice straw and sowing of subsequent wheat with a happy seeder or zero tillage, mulching in other crops, in-situ incorporation; baling and bioenergy generation are available as possible alternatives to burning^8^. Crop residue management is important in determining the distribution and availability of phosphorus in cropped soils. The phytate organic P form accounts for ≥ 50% of the total soil P content. However, this form is unavailable to plants^9,10^. The bioavailability of organic P is greatly affected by the activities of soil enzymes, particularly phosphatase enzymes, and is balanced through residue decomposition and microbial immobilization processes. Although the information on the nutrient requirement in the rice–wheat system under conventional sowing is available but the same is lacking under long-term residue management conditions. Therefore, the present research work aimed to identify the effects of crop residue on rice–wheat system productivity and the interaction of crop residue management approaches with the applied P-fertilizer as crop productivity is often influenced by fertilizer application. As there is relatively little information available on the optimum dose of phosphorus fertilizer under long-term residue-treated soils and feasible savings, the outcome of this study can be useful to ascertain the most optimum fertilizer dosage. Therefore, keeping this in mind the present study was planned to study the interactive effect of long-term crop residue management and phosphorus levels on productivity and phosphorus requirement of wheat and soil health in rice–wheat systems.

## Materials and methods

### Experimental site description

A long-term field experiment was initiated on the rice–wheat system at a Research farm, Department of Agronomy, Punjab Agricultural University, Ludhiana (30°54' N, 75°48' E, 247 m average mean sea level), India, in 2008 to know the impact of crop residue management on productivity of rice–wheat cropping system. Considering the long-term crop residue management experiment the initial chemical properties and nutrient content of the soil samples has been presented as Supplementary Table 1. For the present study, the soil samples were randomly taken before the establishment of the P-level sub-plot experiment (0–15 cm) with a screw auger to study basic soil chemical and physical properties. The soil of the experimental site belonged to Typic Ustochrept, was sandy loam in texture with normal pH and electrical conductivity while exhibiting low soil organic carbon and medium available N, P, and K contents (Table 1).

**Table 1:** The physical and chemical properties of the experimental soil.

Property	Values (units)
Soil texture	Sandy loam [Sand: 69.81%, silt: 18.12%, clay: 12.07%]
Soil pH (1:2.5)	7.16
Electrical conductivity	0.20 dS m^–1^
Soil organic carbon	0.33%
Available N	127.29 mg kg^–1^
Available P	8.84 mg kg^–1^
Available K	111.61 mg kg^–1^

### Experimental set-up

In 2008, a field experiment was established consisting of six crop residue management practices/planting techniques to study the long-term effect of tillage and residue management on crop yields in rice (*Oryza sativa* L.) and wheat (*Triticum aestivum* L.). The same experiment was modified after 11 years and continued for two more consecutive years (2019–20 and 2020–21), consisting of six crop residue management treatments as main plots. The subplots included three phosphorus levels viz., 0, 30 and 60 kg P_2_O_5_ ha^–1^ to study the interactive effect of long-term crop residue management and phosphorus levels on productivity and P-requirement of wheat and soil health in rice–wheat system. The experiment was conducted in a split-plot design with three replications. The plot size was 10.0 m^2^ (2.5 m × 4.0 m). The details of various treatments carried out in the summerand winter seasons are given in Table 2. In zero tillage treatments without paddy straw, the sowing was done with a zero till drill machine after removing the straw. However, in zero tillage plots with paddy straw residue, the crop was sown with turbo happy seeder after uniformly spreading the rice residue. In conventional tillage treatments, sowing was done with a conventional seed cum fertilizer drill. In burning treatments, the loose straw was partially burnt, and sowing was done with zero till drill in standing rice stubbles.

**Table 2:** Detail of treatments in summer and winter season.

	Summer	Winter
Main plots: CRM practices^6^		
T1	Transplanted rice (after removing wheat straw)	Zero tillage sowing of wheat (after removing paddy straw)
T2	Transplanted rice (after removing wheat straw)	Conventional tillage sowing of wheat (after removing paddy straw)
T3	Transplanted rice (after removing wheat straw)	Conventional tillage sowing of wheat (with paddy straw)
T4	Transplanted rice (after removing wheat straw)	Zero tillage sowing of wheat (with paddy straw)
T5	Transplanted rice (with wheat straw)	Zero tillage sowing of wheat (with paddy straw)
T6	Transplanted rice after burning of wheat straw	Zero tillage wheat after partial burning of paddy straw
Subplots: phosphorus levels^3^		
P0	0 kg P_2_O_5_ ha^–1^	
P1	30 kg P_2_O_5_ ha^–1^	
P2	60 kg P_2_O_5_ ha^–1^	

### Crop management

In the summer season, rice variety PR 127 was transplanted in the second fortnight of June with a spacing of 20 cm × 15 cm during both years of study. The nitrogen (125 kg ha^–1^) to rice was applied through urea in three equal splits at 1, 3 and 6 weeks after transplanting. The crop was harvested in the first week of October for both years. In the winter season, sowing of high-yielding wheat variety *Unna*t PBW 343 was done in Ist week of November using seed (100 kg ha^–1^) following row-to-row spacing of 22.5 cm during both years of study. Single super phosphate (16% P_2_O_5_) was used as a phosphorus fertiliser source, and a dose of phosphorus was applied per treatment. Fertilizer N (120 kg N ha^–1)^ was applied through urea (46% N) and potassium (30 kg K_2_O ha^–1^) through muriate of potash (60% K_2_O) uniformly to all the treatment plots. Nitrogen to wheat was applied at sowing, crown root initiation (CRI) stage and maximum tillering in equal splits. The P and K-fertilizers were applied as basal soil doses by drilling the field soil at sowing. For root-damaging insect pests, particularly termite management, the seeds were treated with Chlorpyriphos 20 EC (4 ml per kg seed). For weed management, clodinafop (Topik 15 WP) at the rate of 400 g ha^–1^ and metsulfuron (Algrip 20 WP) (25 g ha^–1^) were sprayed at 30–35 days after sowing by tank mixing in 375 L of water. Irrigations (75 mm depth each) were applied at four critical stages; CRI, late tillering, booting and milking stages. Two sprays of Tilt 25 EC (propiconazole) (500 ml ha^–1^) at the booting and earing stage to protect the crop from yellow rust and one spray of Rogor 30 EC (dimethoate) (375 ml ha^–1^) at the earing stage for control of aphid were given. The wheat crop harvesting was carried out manually during the month of April in both years of study.

### Agrometeorological data

The climate of the study site is sub-tropical and semiarid with dry and hot summer (April–June), moist weather from July–September and cool and dry winter (November to January). The maximum temperature rises above 47 °C during summer, and the minimum temperature falls below 1 °C during winter in January due to frosty spells. The average annual rainfall during the two years under study was 705 mm. About 75% of annual rainfall is received during the south western monsoon from July to September, and the least rainfall is received in December-January or late spring. During cropping season (winter 2019–20), mean weekly maximum and minimum temperatures ranged from 10.3 to 35.5 °C and 4.9 to 18.4 °C, while respective values ranged from 14.0 to 36.1 °C and 3.5 to 17.9 °C during 2020–21 (Fig. S1).

### Reagent used in the experiments

The analytical grade chemicals were used for the various soil chemical analysis studies and included potassium dichromate, sulphuric acid, ammonia, sodium hydroxide, potassium permanganate, sodium bicarbonate, ascorbic acid, ammonium molybdate, ammonium acetate, and acetic acid. The reagents were used for the microbial viable cell count studies included the prepared media as nutrient agar, actinobacteria agar, fungal agar were procured from HiMedia Laboratories Pvt. Ltd., Mumbai, Maharashtra, India. Analytical grade chemicals were used for the enumeration of the soil enzyme activities and included glucose, methanol, urea, 2,3,5-triphenyltetrazolium chloride,triphenylformazan, p-nitrophenyl phosphate, potassium chloride, and phenyl mercuric acetate.

### Data collection and analysis

#### Soil physicochemical properties

##### Methods used

The soil was sampled in triplicate from 0 to 15 cm soil depth from each plot after harvesting of wheat crop in 2020–21 to know the impact of tillage and crop residue management and phosphorus levels on the soil properties. These samples were appropriately processed, including drying, grinding, and sieving before the analysis. Soil pH and EC (1:2 aqueous suspension)^11^, organic carbon (%)^12^, available N^13^, P^14^ and K^11^ were determined. To determine bulk density, the undisturbed soil samples were taken from two sites in each plot from middle position of 0–15 cm soil depth after 2 year of the study, by using stainless steel cores (5 cm diameter and 5 cm height), soil was then transferred in moisture boxes and dried at 105 °C in an oven till the constant weight was achieved^15^. A double-ring infiltrometer was used to measure the infiltration rate from two sites in each treatment after two years of study. The reading on the depth of water infiltrated into the soil with time was recorded from the inner ring until the water intake rate became constant. The infiltration rate was calculated and expressed in cm hour^–1^^16^. Aggregate analysis to determine the size distribution of aggregates was carried out by using a wet sieving technique as per^17^. Large soil clods collected from 0 to 15 cm using a spade were dehydrated in the shade and crumbed into small aggregates along a natural cleavage using gentle strokes. The aggregates, which passed through an eighty mm sieve, were taken and passed through a 4 mm sieve. The aggregates collected on the 4 mm sieve were utilized to four sets of five sieves each (12.7 cm wide and 5 cm high). The sieve pores ranged in size from 2.0, 1.0, 0.5, 0.25, and 0.1 mm. Over the top sieve of the set, the soil peds were equally dispersed, and the capillary was moistened for approximately 10 min. After that, for 30 min, the sieve set was stirred. The sieves were then dried in an oven at 105 °C until the constant weight was achieved. The water stable aggregates (WSA) were evaluated by the formula below:

1 $$\mathrm{WSA }>0.25 {\text{mm}}\left(\mathrm{\%}\right)=\frac{\sum_{i=1}^{n}{w}_{i}}{\mathrm{weight \,\,of \,\,soil\,\, peds}}\times 10,$$

where *n* is the Number of size fractions, *d*_*i*_ is the average diameter for each size range, and *w*_*i*_ is the weight of aggregates in a particular size range as a ratio of the total dry weight of the soil peds taken.

#### Soil biological properties

##### Methods used

For analysing the soil biological properties, soil samples were taken from three spots in each plot after harvest of the crop. The total viable counts of soil bacteria, actinobacteria, and fungi were enumerated using standard serial dilution plate assay on different agar-based media. For serial dilution, 10 g soil samples were added to 90 mL of sterile water blank and shaken for 10 min at 120 rpm. From this 10^–1^ dilution, a series of dilutions were made up to 10^–6^. For enumerating total viable count bacterial count, actinobacteria and fungi 10^–6^, 10^–5^ and 10^–3^ dilutions were used, respectively. The Petri plates were incubated for 2 to 6 days at 28 ± 2 °C in a BOD incubator. The colonies were enumerated and expressed in dry soil per gram as colony-forming units (CFU).

The dehydrogenase enzyme activity was enumerated for the soil samples^18^. One gram of soil sample was incubated with 0.2 mL of TTC (2, 3, 5-triphenyl tetrazolium chloride, 3% w/v) and 0.5 mL of glucose solution (0.5 mL, 1% w/v). The samples were incubated at 28 ± 2 °C for 24 h and, after that, extracted with 10 mL methanol under mild shaking conditions and filtrated using Whatman No. 1. The extract was collected, and absorbance measurements (λ = 485 nm) against the methanol blank were obtained. The final values were calculated through TPF standard curve.

The soil alkaline phosphatase activity was determined^19^ for the soil samples (1 g sample each). The yellow colour formed from the p-nitrophenyl phosphate solution was measured at 420 nm and expressed as µg p-nitrophenol released g^–1^ soil h^–1^.

The urease activity was determined by Bremner and Douglas^20^, in which 5 g of soil sample was taken in a volumetric flask and to this, 5 mL 2000 ppm urea solution was added. The moisture was maintained at 50% WHC and incubated at 37 for 7 h. To this, 2MKCl-PMA is added, and the solution is kept on a shaker for 60 min and filtered. Thereafter, extracting reagent (10 mL) and colouring agents (30 mL) are added, kept in the hot water bath for 30 min, cooled to room temperature and finally, the intensity of the red colour is measured at a wavelength of 527 nm. The enzyme activity is calculated from the standard curve, and the activity is expressed as µg urea/g dry soil/min.

#### Yield attributes and yield of wheat

Effective tillers data was taken from one-meter row length at two locations in each plot at harvest, averaged and then converted into number of effective tillers per m^2^. Spike length was measured from five randomly selected spikes per plot, averaged and expressed in cm. Five randomly selected spikes from each plot was threshed manually, and their grains were counted and averaged for the number of grains per spike. A random sample of grains was drawn from the produce of each plot, and 1000 grains were counted, weighed, and expressed as a 1000-grain weight (g). The bundle weight of each net plot (4 m^2^) was taken before threshing and was expressed as biological yield (t ha^–1^). After threshing, the grain weight of each net plot was measured and converted into grain yield per hectare. The straw yield for each treatment was calculated by subtracting grain weight from bundle weight and expressed as straw yield in t ha^–1^.

#### Statistical analysis

The data was analyzed through Two-way ANOVA, and treatment means were separated by Duncan's multiple range test and compared by least significant difference (LSD)at p ≤ 0.05 using SPSS version 16.0 (SPSS Inc., Chicago, USA) packages.

### Ethics

All the authors abide by the IUCN Policy Statement on Research Involving Species at Risk of Extinction and the Convention on the Trade in Endangered Species of Wild Fauna and Flora.

## Results

### Soil chemical properties

#### Available soil nutrient (N, P, K) content

**Figure 1:**
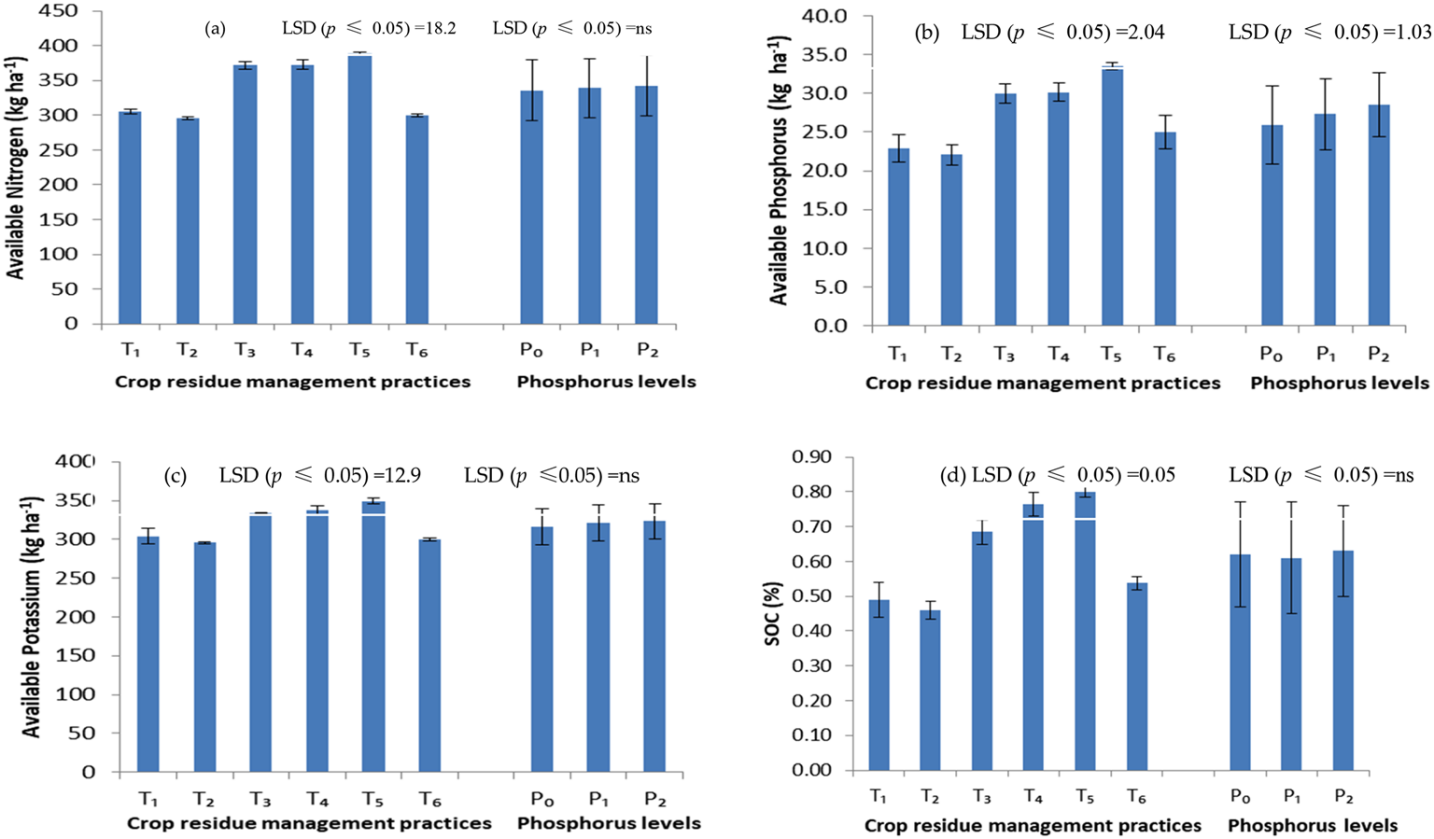
Effect of crop residue management practices and phosphorus levels on chemical properties of soil (**a**) available soil nitrogen, (**b**) available soil phosphorus, (**c**) available soil potassium and (**d**) soil organic carbon at harvest of wheat in the rice–wheat system (pooled data of 2 years). T1: Transplanted rice (After removing wheat straw) (summer season) and zero tillage sowing of wheat (After removing paddy straw) (winter season); T2: Transplanted rice (After removing wheat straw) (summer season) and conventional tillage sowing of wheat (After removing paddy straw); T3: Transplanted rice (After removing wheat straw) (summer season) and conventional tillage sowing of wheat (With paddy straw); T4: Transplanted rice (After removing wheat straw) (summer season) and zero tillage sowing of wheat (With paddy straw) (winter season); T5: Transplanted rice (With wheat straw) (summer season) and zero tillage sowing of wheat (With paddy straw) (winter season); T6: Transplanted rice after burning wheat straw (summer season) and zero tillage wheat after partial burning of paddy straw (winter season). P0: 0 kg P_2_O_5_ ha^–1^; P1: 30 kg P_2_O_5_ ha^–1^; P2: 60 kg P_2_O_5_ ha^–1^.

The data of available soil nutrients (N, P, K) presented in Fig. 1a–c revealed that different crop residue management practices significantly affected the availability of N, P and K in soil. Significant improvement in the availability of nutrients (N, P, K) was found with residue retention/incorporation as compared with residue removal or burning. The available nutrients in the topsoil layer fluctuate from 299.3 to 388 kg ha^–1^ available N, 22.1 to 33.5 kg ha^–1^ available P and 295.7 to 349.3 kg ha^–1^ available K. However, the highest values of available N, P and K of 389.1 kg ha^–1^, 33.5 kg ha^–1^ and 349.3 kg ha^–1^, respectively, were registered with T_5_ treatment where the residue of both crops was retained, and it was significantly higher than conventional practice (T_2_) or residue burning (T_6_) treatments. The various doses of phosphorus did not significantly affect the availability of N and K. The availability of P increased with phosphorus application, and higher P availability was observed with 60 kg P_2_O_5_ ha^–1^.

#### Soil organic carbon

Soil organic carbon content increased with crop residue retention/incorporation treatments irrespective of tillage (Fig. 1d). The soil organic carbon in zero tillage with residue retention treatment was 38.7 and 42.5% higher than in zero tillage without residue (T_1_) and conventional tillage without residue treatment (T_2_). The highest soil organic carbon content was observed under conservation tillage with residue retention treatments, and it was significantly higher than conventional sown wheat without residue or residue burnt treatments.

The perusal of data revealed that varied phosphorus levels had not significantly affected the soil's organic carbon content. However, a numerically higher value was found when a higher dose of phosphorus was applied. It might be due to the higher dry matter production and root mass produced by crops at higher phosphorus levels. The interactions between crop residue management practices and phosphorus levels were found to be non-significant.

### Soil physical properties

#### Soil aggregation

Treatments T_4_ andT_5_, having conservation tillage along with residue retentions, had significantly higher macro-aggregates (8.0–4.75 mm and 4.0–2.0 mm size) as compared to traditional tillage treatments (T_2_) with residue removal (Table 3). This confirms that conservation tillage favours improvement in soil structure by protecting them against destruction or binding the micro-aggregates.

**Table 3:** Effect of crop residue management practices on soil physical properties in the rice–wheat system (pooled data of 2 years).

Treatments	Soil bulk density (g cm^–3^)	Steady-state infiltration rate (cm hr^–1^)	Aggregate size distribution		
CRM practices	0–15 cm		8.00‒4.75 (mm)	4.00‒2.00 (mm)	0.106‒0.25 (mm)
T_1_	1.40^a^	1.8^b^	10.1^b^	9.3^c^	19.8
T_2_	1.42^a^	1.4^c^	9.5^b^	8.9^dc^	21.2
T_3_	1.35^c^	1.5^c^	11.9^ab^	9.5^c^	18.2
T_4_	1.36^c^	2.1^a^	14.1^a^	11.9^a^	16.4
T_5_	1.34^c^	2.2^a^	15.5^a^	12.3^a^	15.8
T_6_	1.39^ab^	1.8^b^	10.2^b^	10.1^b^	19.5
LSD (*p* ≤ 0.05)	0.02	0.1	3.9	0.05	NS

T1: Transplanted rice (After removing wheat straw) (summer season) and zero tillage sowing of wheat (After removing paddy straw) (winter season); T2: Transplanted rice (After removing wheat straw) (summer season) and conventional tillage sowing of wheat (After removing paddy straw); T3: Transplanted rice (After removing wheat straw) (summer season) and conventional tillage sowing of wheat (With paddy straw); T4: Transplanted rice (After removing wheat straw) (summer season) and zero tillage sowing of wheat (With paddy straw) (winter season); T5: Transplanted rice (With wheat straw) (summer season) and zero tillage sowing of wheat (With paddy straw) (winter season); T6: Transplanted rice after burning wheat straw (summer season) and zero tillage wheat after partial burning of paddy straw (winter season). P0: 0 kg P_2_O_5_ ha^–1^; P1: 30 kg P_2_O_5_ ha^–1^; P2: 60 kg P_2_O_5_ ha^–1^.

Different letters indicate significant differences between treatments at Duncan´s test at 5% significance.

#### Soil bulk density

Prolonged application of crop residue in intensive rice–wheat cropping systems had a significant effect on bulk density. Under both zero and conventional tillage, residue retention/incorporation for 11 years caused a significant reduction in bulk density compared to residue removal. At 0–15 cm soil depth, bulk density recorded in zero tillage treatments was lower than in tilled treatments. The lowest bulk density (1.34 g cm^–3^) was found in the T_5_ treatment, where zero tillage was followed along with the retention of residue, and the highest bulk density (1.42 g cm^–3^) was found in T_2_, where no crop residue was retained where conventional tillage was followed (Table 3).

#### Infiltration rate (IR)

The steady-state IR recorded at wheat harvest was significantly affected by different crop residue management practices (Table 3). It was consistently higher with an average of 2.2 cm hr^‒1^ in zero tillage wheat with residue retention treatments (T_5_) compared with an average value of 1.4 cm hr^‒1^ in T_2_ (conventional tillage treatment without residue). This might be due to less soil compaction and more porosity created by the retention of residue on the surface and the burrowing activity of some species. The improved infiltration rate in the Zero tillage + residue retention treatment might have improved the earthworm (surface-feeder group) activity.

### Soil biological properties

#### Microbial count

Microbial activity is the driving force behind the decomposition processes in the soil. The total viable count indicates the overall viable microbial count in the soil. The highest total viable count (59.6 × 10^6^ CFU g^–1^ of dry soil) was obtained in treatment having both wheat and rice residue retained, followed by Zero tillage + residue retention treatment (49.7 × 10^6^ CFU g^–1^ of dry soil) and conventional till wheat with rice residue incorporation (49.5 × 10^6^ CFU g^–1^ of dry soil). The zero tillage (37.9 × 10^6^ CFU g^–1^ of dry soil) and conventional tillage (33.2 × 10^6^ CFU g^–1^ of dry soil) without residues showed comparatively lower total viable count. A significantly lower total viable count (36.7 × 10^6^ CFU g^–1^ of dry soil) was observed in the treatment involving the burning of the residues (Table 4).

**Table 4:** Effect of crop residue management practices and phosphorus levels on soil biological properties in the rice–wheat system (pooled data of 2 years).

Treatments	Microbial activity (0–15 cm)					
	Microbial count (CFU g^–1^ of dry soil)			Enzyme activity		
	Fungi (× 10^3^)	Actinobacteria (× 10^5^)	Total aerobic bacteria (× 10^6^)	Dehydrogenase (µg TPF g^–1^ soil day^-1^)	Alkaline phosphatase (µg pnp released g^-1^soil h^–1^)	Urease (µg urea g^–1^ dry soil min^–1^)
CRM practices						
T_1_	25.9^c^	33.7^d^	37.9^c^	4.29^b^	25.2^c^	3.63
T_2_	23.3^c^	33.9^d^	33.2^c^	4.27^b^	25.5^c^	3.59
T_3_	36.7^b^	44.2^c^	49.5^b^	5.11^b^	31.2^b^	4.43
T_4_	44.8^b^	55.0^b^	49.7^b^	6.34^a^	33.0^b^	4.61
T_5_	53.2^a^	64.0^a^	59.6^a^	6.70^a^	38.5^a^	4.88
T_6_	19.8^c^	32.3^d^	36.7^c^	4.67^b^	25.9^c^	3.62
LSD (*p* ≤ 0.05)	7.9	6.2	7.8	0.90	3.2	NS
Phosphorus levels						
P_0_	33.5	43.8	42.2	4.69^c^	24.5^b^	4.01
P_1_	32.7	43.6	45.0	5.10^b^	32.1^a^	4.27
P_2_	35.5	44.1	46.0	5.89^a^	33.1^a^	4.09
LSD (*p* ≤ 0.05)	NS	NS	NS	0.60	2.3	NS
Interaction	NS	NS	NS	NS	NS	NS

T1: Transplanted rice (After removing wheat straw) (summer season) and zero tillage sowing of wheat (After removing paddy straw) (winter season); T2: Transplanted rice (After removing wheat straw) (summer season) and conventional tillage sowing of wheat (After removing paddy straw); T3: Transplanted rice (After removing wheat straw) (summer season) and conventional tillage sowing of wheat (With paddy straw); T4: Transplanted rice (After removing wheat straw) (summer season) and zero tillage sowing of wheat (With paddy straw) (winter season); T5: Transplanted rice (With wheat straw) (summer season) and zero tillage sowing of wheat (With paddy straw) (winter season); T6: Transplanted rice after burning wheat straw (summer season) and zero tillage wheat after partial burning of paddy straw (winter season). P0: 0 kg P_2_O_5_ ha^–1^; P1: 30 kg P_2_O_5_ ha^–1^; P2: 60 kg P_2_O_5_ ha^–1^.

*FUN* fungi, *ACT* actinobacteria, *TAB* total aerobic bacteria, *DHA* dehydrogenase, *PHOS* phosphatase and *URE* urease.

Different letters indicate significative differences between treatments at Duncan´s test at 5% significance.

The highest fungal activity (53.2 × 10^3^ CFU g^–1^ of dry soil) was obtained in treatment having both wheat and rice residue retained, followed by Zero tillage + residue retention treatment (44.8 × 10^3^ CFU g^–1^ of dry soil) and conventional till wheat with rice residue incorporation (36.7 × 10^3^ CFU g^–1^ of dry soil). The zero tillage (25.9 × 10^3^ CFU g^–1^ of dry soil) and conventional tillage (23.3 × 10^3^ CFU g^–1^ of dry soil) without residues showed comparatively lower fungal count. The lowest fungal count (19.8 × 10^3^ CFU g^–1^ of dry soil) was observed in the residue-burning treatment. The decline in fungal count could be attributed to the burning practice.

Actinobacteria play a very important role in the degradation of crop residues. The highest actinobacterial activity (64.0 × 10^5^ CFU g^–1^ of dry soil) was obtained in treatment having both wheat and rice residue retained, which was statistically significant as compared to the actinobacterial count in burning practice (32.3 × 10^5^ CFU g^–1^ of dry soil). Comparatively higher actinobacterial count (55.0 and 44.2 × 10^5^ CFU g^–1^ of dry soil) was observed in treatments receiving crop residues (T_4_ and T_3_) however, less actinobacterial count (33.9 and 33.7 × 10^5^ CFU g^–1^ of dry soil) was found in treatments without residue retention (T_2_ and T_1_).

#### Microbial enzyme activities

Enzyme activity in soil is an indirect measure of different microbial processes going on in the soil. The highest dehydrogenase activity (6.7 µg TPF g^–1^ soil day^–1^) was observed in zero till crop residue retention treatment (rice and wheat residue retention), followed by 6.34 µg TPF g^–1^ soil day^–1^ in zero till rice residue retention. Similarly, the highest phosphatase activity (38.5 µg p-nitrophenol released g^–1^ soil h^–1^) was observed in zero till crop residue retention treatment (rice and wheat residue retention), followed by 33.0 µg p-nitrophenol released g^–1^ soil h^–1^ in zero till rice residue retention. Urease activity was highest for rice and wheat residue retention using zero till (4.88 µg urea/g dry soil/min) followed by zero (4.61 µg urea g^–1^ dry soil min^–1^) and conventional till with rice residue retention (Table 4).

### Yield attributes and yield of wheat

The crop residue management practices and graded phosphorus doses significantly affected the yield attributes and yield in wheat. The effective tillers of wheat under residue of rice alone or both rice and wheat were not significantly affected by phosphorus levels. However, the highest effective tillers had the highest phosphorus level (P_2_). However, maximum effective tillers under treatment (T_1_ and T_2_) where wheat was sown without crop residue were obtained with P_2_ (60 kg P_2_O_5_ ha^–1^), significantly higher than P_0_ and P_1_. Maximum spike length (11.6 cm) was recorded in the T_5_ treatment, which was at par with T_3_ and T_4_ but significantly higher than all other treatments. The improvement in spike length where both residues were retained was 11.5% compared to without residue treatments. The test weight (1000-grain weight) was not significantly differed among various treatments, but numerically higher test weight was reported where residue was retained or incorporated (Table 5).

**Table 5:** Effect of crop residue management practices and phosphorus levels on yield attributes and yield of wheat in the rice–wheat system (pooled data of 2 years).

Treatments	Effective tillers (No. m^–2^)	Spike length (cm)	Grains spike^–1^ (No.)	1000-grain weight (g)	Straw yield (t ha^–1^)	Biological yield (t ha^–1^)
CRM practices						
T_1_	286.4^c^*	10.2^b^	43.6^c^	39.6	7.02^c^	12.23^c^
T_2_	290.3^c^	10.4^b^	43.7^c^	39.4	7.18^c^	12.49^c^
T_3_	309.9^b^	11.2^a^	49.2^ab^	40.0	7.67^b^	13.51^b^
T_4_	307.3^b^	11.2^a^	48.5^b^	40.3	7.56^b^	13.45^b^
T_5_	333.0^a^	11.6^a^	50.9^a^	40.6	7.93^a^	141.2^a^
T_6_	295.3^c^	10.4^b^	45.2^c^	39.4	7.27^c^	12.7.9^c^
LSD (p ≤ 0.05)	11.3	0.5	2.6	NS	0.38	0.58
Phosphorus levels						
P_0_	287.4^c^	10.5^b^	44.8^c^	39.7	7.11^c^	12.38^c^
P_1_	306.3^b^	10.9^a^	47.2^b^	39.9	7.45^b^	13.18^b^
P_2_	317.5^a^	11.1^a^	48.6^a^	40.0	7.75^a^	13.72^a^
LSD (p ≤ 0.05)	6.3	0.2	1.1	NS	0.24	0.26
CRM x P	NS	NS	NS	NS	NS	NS

T1: Transplanted rice (After removing wheat straw) (summer season) and zero tillage sowing of wheat (After removing paddy straw) (winter season); T2: Transplanted rice (After removing wheat straw) (summer season) and conventional tillage sowing of wheat (After removing paddy straw); T3: Transplanted rice (After removing wheat straw) (summer season) and conventional tillage sowing of wheat (With paddy straw); T4: Transplanted rice (After removing wheat straw) (summer season) and zero tillage sowing of wheat (With paddy straw) (winter season); T5: Transplanted rice (With wheat straw) (summer season) and zero tillage sowing of wheat (With paddy straw) (winter season); T6: Transplanted rice after burning wheat straw (summer season) and zero tillage wheat after partial burning of paddy straw (winter season). P0: 0 kg P_2_O_5_ ha^–1^; P1: 30 kg P_2_O_5_ ha^–1^; P2: 60 kg P_2_O_5_ ha^–1^.

*Different letters indicate significant differences between treatments at Duncan´s test at 5% significance. Means followed by the same letter or without letter in each column are not significantly different at P = 0.05.

Spike length and grains per spike were significantly affected by various phosphorus levels. The highest was registered with the highest phosphorus level (60 kg P_2_O_5_ ha^–1^), significantly higher than the control treatment (0 kg P_2_O_5_).

Maximum grain yield (6.19 t ha^–1^) was registered in T_5,_ where wheat was sown with a happy seeder and residue of both wheat and rice crops was retained significantly higher than residue removal or burning treatments (Table 6). The improvement in grain yield in T_5_ was 8.6 and 7.6% over T_1_ and T_2_ (without residue) while 5.7% over T_6_ (partial burning). The interaction effect of crop residue management practices and phosphorus levels on grain yield was significant (Table 6). In T_5_, where the residue of both the crops was retained, gave similar yields at 0, 30 and 60 kg P_2_O_5_ ha^–1^. In the treatments where the residue of a single crop was retained/ incorporated (T_3_ and T_4_), grain yield obtained with P_1_ and P_2_ was statistically at par but significantly higher than P_0_. However, without residue treatments (T_1_ and T_2_), the highest grain yield was obtained when 60 kg P_2_O_5_ ha^–1^ was applied, significantly higher than 30 kg P_2_O_5_ ha^–1^ and control (No P_2_O_5_) treatment. Maximum straw and biological yield were obtained in the T_5_ treatment, which was significantly higher than other crop residue management treatments. The straw yield was 12.9 and 9.1% higher, where the residue of both rice and wheat crop was retained as compared to conventional tillage sown wheat with residue removal (T_2_) and residue burning treatment (T_6_). Phosphorus doses also significantly influenced the straw and biological yield. The highest straw yield (7.27 t ha^–1^) was registered when 60 kg P_2_O_5_ ha^–1^ was applied, significantly higher than P_1_ and P_0_ treatments. The biological yield was 10.8 and 4.1% higher, with a higher dose of phosphorus (P_2_) over the P_0_ and P_1_ levels.

**Table 6:** Interactive effect of crop residue management practices and phosphorus levels on grain yield of wheat (pooled data of 2 years).

Treatments	Grain yield (t ha^–1^)		
	Phosphorus levels (kg ha^–1^)		
CRM practices	P_0_	P_1_	P_2_
T_1_	4.62f.	5.25de	5.77bc
T_2_	4.76f.	5.34d	5.84b
T_3_	5.56c	5.95b	6.03ab
T_4_	5.60c	6.01b	6.08ab
T_5_	6.10ab	6.22ab	6.26a
T_6_	5.04e	5.65c	5.87b
LSD (*p* ≤ 0.05) (CRM x P)	0.24		

T1: Transplanted rice (After removing wheat straw) (summer season) and zero tillage sowing of wheat (After removing paddy straw) (winter season); T2: Transplanted rice (After removing wheat straw) (summer season) and conventional tillage sowing of wheat (After removing paddy straw); T3: Transplanted rice (After removing wheat straw) (summer season) and conventional tillage sowing of wheat (With paddy straw); T4: Transplanted rice (After removing wheat straw) (summer season) and zero tillage sowing of wheat (With paddy straw) (winter season); T5: Transplanted rice (With wheat straw) (summer season) and zero tillage sowing of wheat (With paddy straw) (winter season); T6: Transplanted rice after burning wheat straw (summer season) and zero tillage wheat after partial burning of paddy straw (winter season). P0: 0 kg P_2_O_5_ ha^–1^; P1: 30 kg P_2_O_5_ ha^–1^; P2: 60 kg P_2_O_5_ ha^–1^.

Different letters indicate significant differences between treatments as per the mean separation performed through Duncan´s test at 5% significance.

## Discussion

### Soil health

Conservation tillage with residue retention has significantly improved the physicochemical, microbial counts and soil enzyme properties of soil. Conservation tillage increases soil aggregation by decreasing soil disturbance and also by increasing soil organic matter and possibly the growth of microflora, especially fungi, which help to bind soil particles and micro-aggregates together^21^. Significant improvement in mean weight diameter was recorded with residue retention under conservation tillage compared to conventional tillage without residue or residue burnt treatments. Singh et al.^22^ also documented significant improvement in the mean weight diameter of aggregates and aggregate stability in sandy loam soil under rice–wheat cropping system with residue addition due to an increase in soil organic carbon since soil organic matter plays an important role in soil aggregation. Lower aggregation in conventional tillage might be due to heavy machinery and intensive tillage (twice disc, twice cultivators and one planking operation). Lower bulk density was observed in residue retained treatments, i.e. T_5_ (1.34 g cm^–3^) and T_4_ (1.36 g cm^–3^), and the highest was found in conventional tillage with residue removal T_2_ (1.42 g cm^–3^) at 0–15 cm soil depth (Table 1). The lower bulk density in zero-tilled treatments with residue retention might be associated with greater soil biological activities, especially earthworms^23^ Also, the studies of Singh et al.^24^, Alam et al.^25^, Meena et al.^26^ identify the similar results. Several studies have documented higher bulk density under ZT at the surface layer than in tilled soil^27–29^. However, the results of this field study do not comply with earlier reports regarding the lowering of bulk density in ZT + residue retention treatments as compared to the conventional tillage (CT) in the upper soil surface, especially in fine-textured soils, which is ascribed to the development of organic-rich mulch and therefore, a possibly enhanced microbial activity especially earthworms^23^ and higher bulk density or no difference in bulk density in conventional tillage (CT) in the deeper soil layers^30^. The crop residues over the upper soil surface prevent aggregate breakdown by direct raindrop effect and rapid drying and wetting of soils. Various studies show little or no differences in bulk density between conventional tillage (CT) and zero tillage (ZT) in surface soil layers^31^.

Conservation tillage + residue retention improved the infiltration rate over residue removal treatment^32^. The improved aggregate stability and moisture retention could explain the enhanced infiltration rates. Furthermore, the occurrence and sustenance of macrofauna components, particularly the earthworms, through burrowing activities can also be identified as the other possible aspect^33^. Significant improvement in the SOC in the upper soil layer (0–15 cm) in the crop residue management practices was recorded as compared to no residue or residue removal treatments (Fig. 1d). Gupta et al.^34^, Mondal et al.^35^, Fang et al.^36^, Zhang et al.^37^, Zhang et al.^38^, Chen et al.^39^, Liu et al.^40^ and many other researchers reported significant improvement in soil organic carbon under zero tillage with residue retention as compared to conventional practice. Tillage and crop residue management practices improved soil structure associated with the protection of SOM and biological activities^41^, ultimately improving soil aggregation, soil organic matter and nutrient cycling in the rice–wheat system. The increase in the availability of soil nutrients under conservation tillage with residue retention treatments might be due to improved soil health and SOC. Gupta et al.^34^, Bhattachariya et al.^42^, Zhang et al.^38^ and Zhao et al.^43^ also reported an increase in nutrient availability with residue incorporation/retention as compared to residue removal.

Soil microorganism is important in increasing soil quality, health, fertility and soil microbial community. Among all parameters, microbial activity is considered a sensitive indicator for evaluating soil quality. Adding crop residues stimulated the microbial activity of bacteria, fungi and actinomycetes^44^. The findings of these workers corroborate our findings where higher total viable count, fungal count and actinobacterial count were found in treatments receiving crop residues. In an experiment conducted by Pintari et al.^45^, significantly higher soil respiration was observed when straw was incorporated into the top 10 cm compared to the control with no straw added. Higher total phospholipid-derived fatty acids (PLFAs), fungal PLFAs content and increased value of fungi/G^+^ bacteria + G^−^ bacteria under combined application of tillage with crop residue incorporation were observed by Tang et al.^46^. Likewise, another study identified the beneficial impact of residue returning on the soil microbial diversity^47^.

Both microbial counts and enzyme activities were found to be lowest in treatment following a burning practice. This could be due to a reduction in microbial biomass, microbial activity, respiration, and decomposition process and sometimes also alters the composition of the microbial community. Kumar et al.^48^, also reported a comparatively lower bacterial, fungal and actinobacterial count in treatments where paddy straw was burnt. The highest count was observed in the paddy straw retained plots.

The microbial decomposition process is a key component of nutrient geochemical cycles. The microbial diversity and physiological versatility are improved by incorporating or retaining the agri-residue in fields. The agri-residue comprised of complex carbohydrates, proteins and other useful minerals can be acted upon the soil microflora as an energy and nutrient source, favouring improved microbial growth and multiplication rates. Several microbial lytic enzymes released extracellularly by various genera can be measured and compared with no residue amendment treatments. In the present study higher enzyme activities were recorded in the plots amended with residues. Higher enzymatic activity of dehydrogenase and acid and alkaline phosphatase in residue-retained plots than those without residues have also been reported by Choudhary et al.^44^ and Gupta et al.^21^.

### Yield attributes and yield of wheat

Significantly higher wheat grain yield under conservation tillage with residue retention could be due to more favourable conditions created by conservation agricultural practices, i.e. improvement in water conservation, lower bulk density, better soil aggregation and better nutrient recycling due to elevation in soil organic carbon in T_5_ and T_4_ treatments where wheat was sown in standing stubbles of rice with both rice and wheat residue and single rice residue, respectively. Higher grain yield under residue retention/incorporation treatments might be due to increased growth parameters. Yield attributes (effective tillers, spike length, grains per spike, 1000-grain weight) resulted from the increase in soil organic matter content and availability of nutrients and moderation of the hydrothermal regime of the soil^49–51^. Straw incorporation/retention improves the soil properties (physical, chemical and biological), resulting in better root growth and more uptake of nutrients by the plants, ultimately leading to better plant growth and an increase in grain yield. Similar results were also documented by Gupta et al.^49^, Chaudhary and Iqbal^52^, Meena et al.^26^ and Sharma et al.^53^.

## Conclusions

Phosphorus fertilizer dosages for wheat crop are required to be optimized for the crop residue management approaches. The residue retention/incorporation significantly influenced the soil's physical, chemical, and biological properties besides decreasing the phosphorus fertilizer requirements of the crop. The residue management practice exhibited a significant effect on the viable cell counts of all the test microbes and the urease enzyme activity except dehydrogenase and phosphatase activities. Furthermore, this study revealed that the phosphorus levels had a significant effect on grain yield traits of wheat and the P-levels exhibited significant interactions with the different crop residue management practices. The grain yield of wheat was equivalent to the recommended dose of the P-fertilizer (i.e. 60 kg P_2_O_5_ ha^–1^)on retention/incorporation of rice residue alone with application of the half of the recommended dose of P-fertilizer(30 kg P_2_O_5_ ha^–1^). While, incorporation/retention of both rice and wheat residues resulted no need for application of P-fertilizer. Therefore, the crop residue incorporation/retention can help facilitate the adequate P-content for the crop growth and productivity at relatively lower P_2_O_5_ fertilizer doses.

### Supplementary Information

Supplementary Information.

## Data Availability

The datasets used and/or analyzed during the current study are available from the corresponding author on reasonable request.
