# Supplementary material for: Interactive effects of long-term management of crop residue and phosphorus fertilization on wheat productivity and soil health in the rice–wheat
Source: Sci Rep. 2024 Jan 16;14:1399. doi: 10.1038/s41598-024-51399-8 (PMC10791631; doi:10.1038/s41598-024-51399-8)
Supplement: Supplementary file 1 — Supplementary Information. [file 41598_2024_51399_MOESM1_ESM.docx]

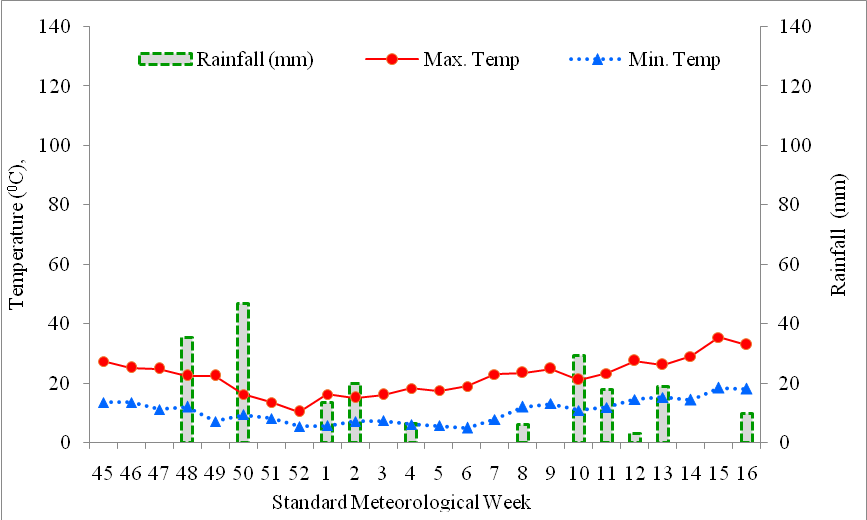


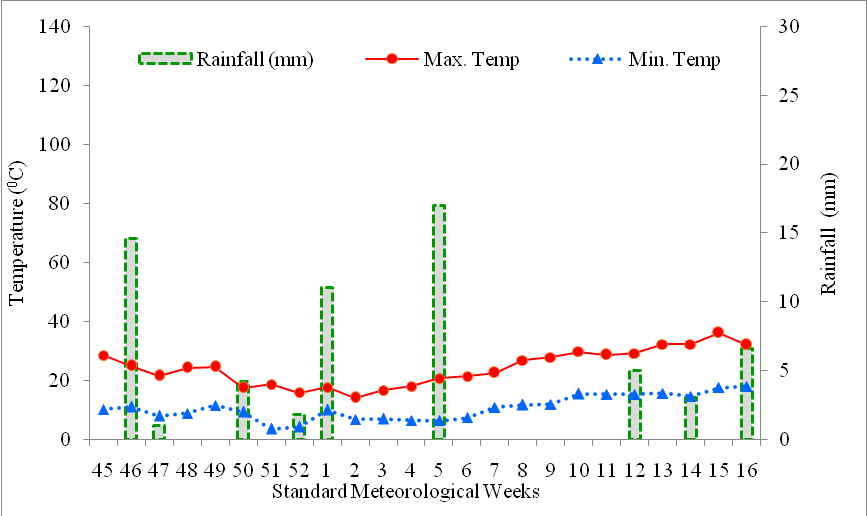


**Figure S1: Weather data during cropping season 2019-20 and 2020-21**

**Supplementary Table 1.** The initial status of soil (0-15 cm) at the start of the main plot agri-residue management treatments in the year 2008

| **Parameter** | **Value (units)** |
| --- | --- |
| Bulk density | 1.43 g cm^-3^ |
| Organic Carbon | 0.33 % |
| Available nitrogen | 285.1 kg ha^-1^ |
| Available phosphorus | 19.8 kg ha^-1^ |
| Available potassium | 250.0 kg ha^-1^ |
